# Bulk Layering Effects of Ag and Cu for Tandem CO_2_ Electrolysis

**DOI:** 10.1002/cssc.202401769

**Published:** 2024-12-03

**Authors:** Mark Sassenburg, H. P. Iglesias van Montfort, Nikita Kolobov, Wilson A. Smith, Thomas Burdyny

**Affiliations:** ^1^ Department of Chemical Engineering Delft University of Technology 2629HZ Delft The Netherlands; ^2^ Department of Chemical and Biological Engineering and Renewable and Sustainable Energy Institute (RASEI) University of Colorado Boulder Boulder, Colorado 80303 United States; ^3^ National Renewable Energy Laboratory Golden 80401 Colorado United States

**Keywords:** Electrochemistry, CO_2_reduction, Silver, Copper, Tandem catalyst

## Abstract

The electrochemical reduction of carbon dioxide (CO_2_) presents an opportunity to close the carbon cycle and obtain sustainably sourced carbon compounds. In recent years, copper has received widespread attention as the only catalyst capable of meaningfully producing multi‐carbon (C_2+_) species. Notably carbon monoxide (CO) can also be reduced to C_2+_ compounds on copper, motivating tandem systems that combine copper and CO‐producing species, like silver, to enhance overall C_2+_ selectivities. In this work, we examine the impact of layered‐combinations of bulk Cu and Ag by varying the location and proportion of the CO‐producing Ag layer. We report an effective increase in the C_2+_ oxygenate selectivity from 23 % with a 100 nm Cu to 38 % for a 100 : 15 nm Cu : Ag layer. Notably, however, for all co‐catalyst cases there is an overproduction of CO vs Cu alone, even for 5 nm Ag layers. Lastly, due to restructuring and interlayer mobility of the copper layer it is clear that the stability of copper limits the locational advantages of such tandem solutions.

## Graphical abstract

**Good on its own**: Silver is frequently proposed in tandem Cu−Ag systems with the idea of enhancing CO supply for C_2+_ product formation. By controlling the Ag−Cu catalyst composition, we studied product distribution and found no improvements in C_2+_ selectivity. These results indicate that the Cu catalyst alone produces a sufficient amount of CO.

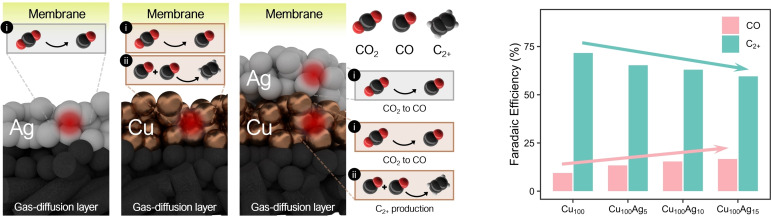

## Introduction

Converting CO_2_ electrochemically in an electrolyzer poses an attractive opportunity to generate fuels and base chemicals in a potentially carbon‐neutral way. Throughout numerous CO_2_ electrolysis (CO_2_RR) studies, research has related the formed products and their selectivity to the used catalysts, producing a wide range of value‐added chemicals.[^1^, ^2^, ^3^, ^4^] Two of the most promising materials with the potential to impact production chains are silver (Ag) and copper (Cu).^[5]^ Ag catalysts are well‐known for effectively converting CO_2_ into carbon monoxide (CO) with near absolute selectivity (90–95 %). The obtained CO can then be combined with hydrogen (H_2_) to be upgraded to longer carbon chains using Fisher‐Tropsch synthesis.^[6]^ Cu catalysts, on the other hand, do not require C1 upgrading to make multi‐carbon products like ethylene (C_2_H_4_) and ethanol (C_2_H_5_OH), due to the unique binding energy of intermediates to its active sites that allow CO to undergo further reduction.[^7^, ^8^] These multi‐carbon products carry individually higher value and utilization potential as chemical building blocks and energy dense fuels. However, the ability for Cu to produce a wide spectrum of products is also problematic for reaching high selectivity towards any given product, leading to excessive downstream separation costs.[^9^, ^10^]

The ability for Cu to produce multi‐carbon products stems from the intermediate binding energy of *CO after being reduced from CO_2_. Such an intermediate binding allows for both the dimerization of two bound *CO surface species through a Langmuir‐Hinshelwood mechanism, but also for the reaction between an aqueous CO species with a surface *CO intermediate.^[11]^ These mechanisms similarly explain why Cu is able to further perform the CO reduction reaction (CORR) with a similar product spectrum but a moderately higher ethanol/acetate‐to‐ethylene ratio.[^12^, ^13^, ^14^, ^15^] To narrow the products formed and increase C_2+_ selectivity, researchers have questioned whether Cu can further profit from excess CO in the reaction medium, or by modulating the binding strength between Cu and surface‐bound intermediates. These considerations have led to the development of bimetallic catalysts that can be grouped into two clusters of design approaches: atomistic and bulk.

In the first approach, what we call the *atomistic* structuring of the catalyst, researchers aim to modulate Cu and its interactions with CO_2_RR intermediates. In these cases, Cu is either doped with a secondary metal, or bimetallic alloy nanoparticle clusters are formed with the intention of altering the local chemical potential by means of d‐band interaction. Looking specifically into Cu−Ag tandem systems, there are numerous examples which modulate overall selectivity. Some cases show an increase in CO activity,^[16]^ control over syngas composition,^[17]^ or improvement of selectivity towards methane (CH_4_),^[18]^ ethylene (C_2_H_4_),[^19^, ^20^] acetate (CH_3_COO^−^), and ethanol (C_2_H_5_OH).[^21^, ^22^, ^23^, ^24^, ^25^] The amount of added Ag can also be used to tune the overall C_2+_‐selectivity.[^26^, ^27^]

In the second approach, the CO_2_RR products formed on Cu are influenced by adding *bulk* co‐catalysts in the vicinity of Cu. A typical example is the addition of Ag or Au metals than can produce CO and spillover to Cu for further reduction. Here *bulk* refers to any added materials that can react with CO_2_ or CO_2_RR by‐products but are not clearly modulating atomistic Cu reactivity directly (e. g. a Cu and Ag nanoparticle next to each other where the interfacial effects are likely negligible). Such a distinction is necessary as bulk approaches can be more predictably engineered through mixing, layering and co‐deposition to achieve a desired outcome than atomistic bimetallic systems. Whereas aforementioned works make use of a potential‐field effect, the incorporation of Ag directly into the bulk can be used to promote the CO_2_‐to‐CO step[^28^, ^29^] by means of spillover, and stabilize the catalytic layer, especially given the inherent instability of copper species.[^30^, ^31^, ^32^, ^33^, ^34^] Besides providing structural integrity, monoatomic Ag in small quantities has been shown to create compressive surface strain in the Cu host lattice, which modified the electronic structure to suppress H_2_ evolution and favor the formation of multi‐carbon oxygenates.^[35]^ While the presented Cu−Ag bimetallic materials are proven to be effective in tuning the product distribution, many of these tests were performed under highly controlled conditions and reaction rates limited to a few mA cm^−2^. This poses the question whether the observed results can be translated one‐to‐one towards high‐rate electrolysis, as needed for scaling of this technology.^[36]^

In recent years, studies on CO_2_RR have shifted towards using gas diffusion electrodes (GDEs) where diffusional length of CO_2_ to catalytic sites is greatly reduced. In these systems both reaction rates (0.1–1 A cm^−2^) and catalytic surface area are greatly increased, and Cu catalysts by themselves can achieve >80 % C_2+_ product selectivity at elevated current densities.[^25^, ^37^] Here by‐product CO in particular decreases at elevated reaction rates, indicating that additional CO availability could be important to fuel further increased C_2+_ current densities. The examples of co‐catalyst additions in GDE systems are less than in the lower current density aqueous systems, but bimetallic Cu−Ag systems have been tested. In one example sequential catalysis was performed, where Ag and Cu catalysts were fully separated into upstream and downstream catalytic sections. Here C_2+_ selectivity was increased to >80 %, but 10 % CO remained in the final product mix.^[38]^ In other GDE bimetallic examples, the produced by‐product CO was always produced in excess, hurting the end selectivity of C_2+_ products.[^23^, ^39^] We then had the question; how much Ag CO‐producing catalyst is too much? And where in a catalytic layer should a CO‐producing catalyst be located?

**Figure 1:**
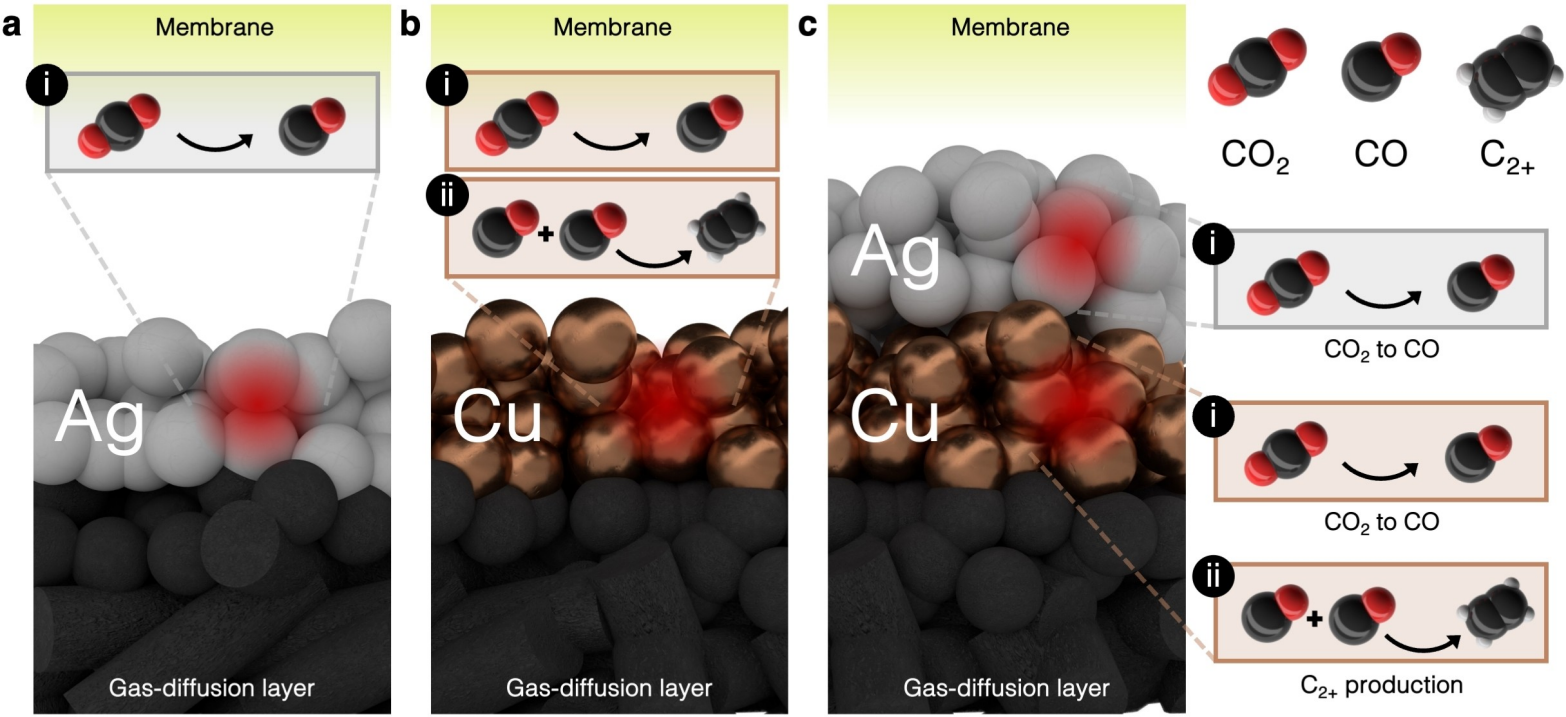
Schematic representation of CO_2_RR mechanisms in systems with different catalysts. (**a**) Pure Ag mainly promotes CO_2_−to−CO conversion (i). (**b**) a pure Cu catalyst, facilitating the CO_2_−to−CO conversion (i) and retaining the formed CO to allow further CO−to−C_2+_ dimerization (ii). (**c**) A sequentially layered Cu−Ag system, where aside of the Cu reactions, the CO_2_‐to‐CO conversion on Ag (i) can provide the Cu catalyst with a richer CO environment, influencing reaction (ii).

In this work we further investigate the tandem catalysis of Ag and Cu in a zero‐gap MEA to determine the ideal amount, location and impact of adding Ag co‐catalysts to Cu. With the knowledge that CO is an intermediate product towards multicarbon products, we assess whether an overall increase in C_2+_‐selectivity will occur through the supply of by‐product CO from silver. Or conversely, is the presence of copper itself enough to maintain sufficientintrinsic dimerization activity. To test these hypotheses, we compare the performance of a pure Ag catalyst to perform CO_2_−to−CO reduction (Figure 1a), a pure Cu catalyst capable of dimerization (Figure 1b) and a range of sequentially layered Cu−Ag systems that perform both functions and possibly benefit from a CO‐enriched environment for further CO‐to‐C_2+_ dimerization on Cu (Figure 1c).

## Results and Discussion

**Figure 2:**
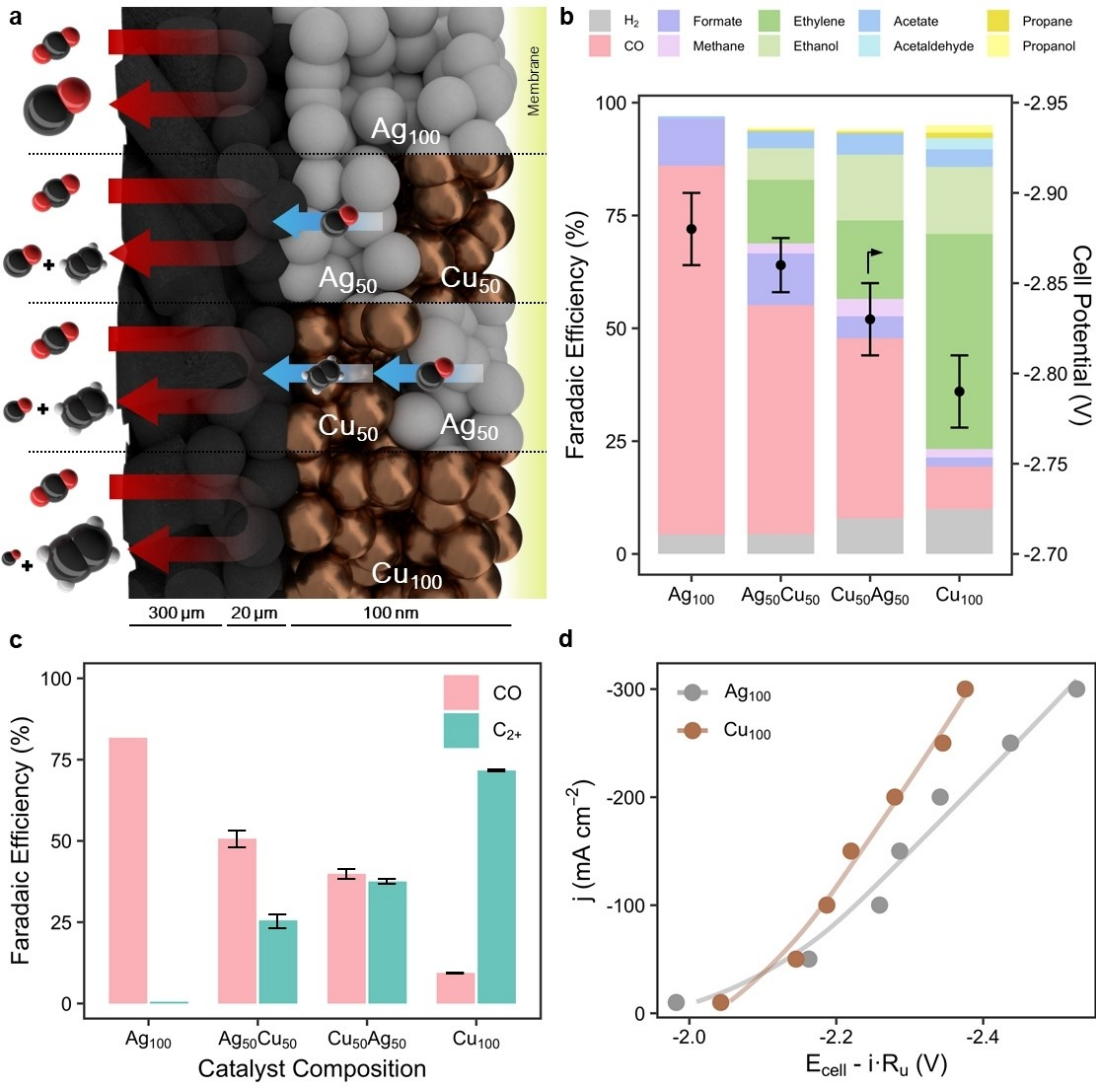
Layering the bulk composition directly affects the selectivity of the cathode. (**a**) Schematic representation of the four different 100 nm catalyst combinations in a zero‐gap membrane‐electrode assembly (MEA). Sizes of the shown product molecules indicate the expected trends in conversion tendency. (**b**) Product distributions of electrochemical conversion at 200 mA cm^−2^ show that for the mixed systems, the material closest to the CO_2_ gas‐liquid interface has a more pronounced effect in selectivity and cell potential. (**c**) A comparison of Faradaic efficiency for CO and C_2+_‐products for the various electrode layer scenarios. (**d**) Polarization curves for Ag_100_ and Cu_100_ samples, corrected for ohmic cell drop. Displayed values are taken from triplicate experiments averaged between minute 5 and 60 at a gas product sample rate of 5 min^−1^. Error bars represent a standard deviation.

As a starting point in assessing performance of tandem layered GDEs, a range of Cu and Ag catalysts were synthesized through sequential sputtering. We first deposited four different compositions for the catalyst layer on gas‐diffusion layers (GDLs), as shown in Figure 2a: firstly, a 100 nm Ag (Ag_100_), followed by 50 nm Ag coated with 50 nm Cu (Ag_50_Cu_50_), 50 nm Cu coated with 50 nm Ag (Cu_50_Ag_50_) and finally a 100 nm Cu layer (Cu_100_). In designing these test, we aimed to maintain the overall catalyst layer thickness to decouple the observed effects in selectivity from the diffusional depth of CO_2_ in the electrolyte.^[40]^ The characterization of samples can be found in the Supporting Information. After deposition of the catalysts, we performed CO_2_RR for each of the catalysts at a fixed current density of −200 mA cm^−2^, which represented the maximum reaction rate for C_2+_‐product formation for Cu at the applied flow rate. The effluent gas‐stream was analyzed every 5 minutes using an in‐line automated gas‐chromatograph (GC).^[41]^

The effects of bulk catalyst layer composition are detailed in Figure 2b and Supporting Table S1. While the pure silver and pure copper electrodes behave as previously reported, the layer solutions show a hybrid selectivity. While CO is still the dominant product for the Ag_50_Cu_50_ and Cu_50_Ag_50_ cases, C_2+_‐hydrocarbons show an increasing Faradaic efficiency (FE) for the Cu_50_Ag_50_ case. A closer look into the differences between the two bimetallic systems reveals that CO selectivity decreases from ~50 % to ~40 % and the C_2+_ selectivity increases from ~26 % to ~38 % for Ag_50_Cu_50_ and Cu_50_Ag_50_, respectively (Figure 2c.). Additionally, the potential required to run both tandem catalysts at −200 mA cm^−2^ appears to follow a similar trend where Cu_50_Ag_50_ performs closer to Cu_100_ and Ag_50_Cu_50_ to Ag_100_. There are, however, some non‐linear effects in this series, like the slight increase in methane (CH_4_) and acetate (CH_3_COO^−^) for the for Cu_50_Ag_50_ case as compared to Cu_100_.

The primary conclusion from Figure 2, however, is the clear preference for the Ag catalyst to be positioned on the membrane side of the catalyst (Cu_50_Ag_50_). The results highlight that the CO produced on the Ag catalyst must diffuse past the Cu layer, and thus can be further reduced. The amount of CO produced for both Cu_50_Ag_50_ and Ag_50_Cu_50_, however, is substantial versus the pure copper case. Alongside the knowledge that only Cu can effectively dimerize CO molecules, this implies an equivalent ratio of Cu and Ag will result in an overproduction of CO that escapes to the gas channel and thus does not benefit C_2+_ selectivity.

**Figure 3:**
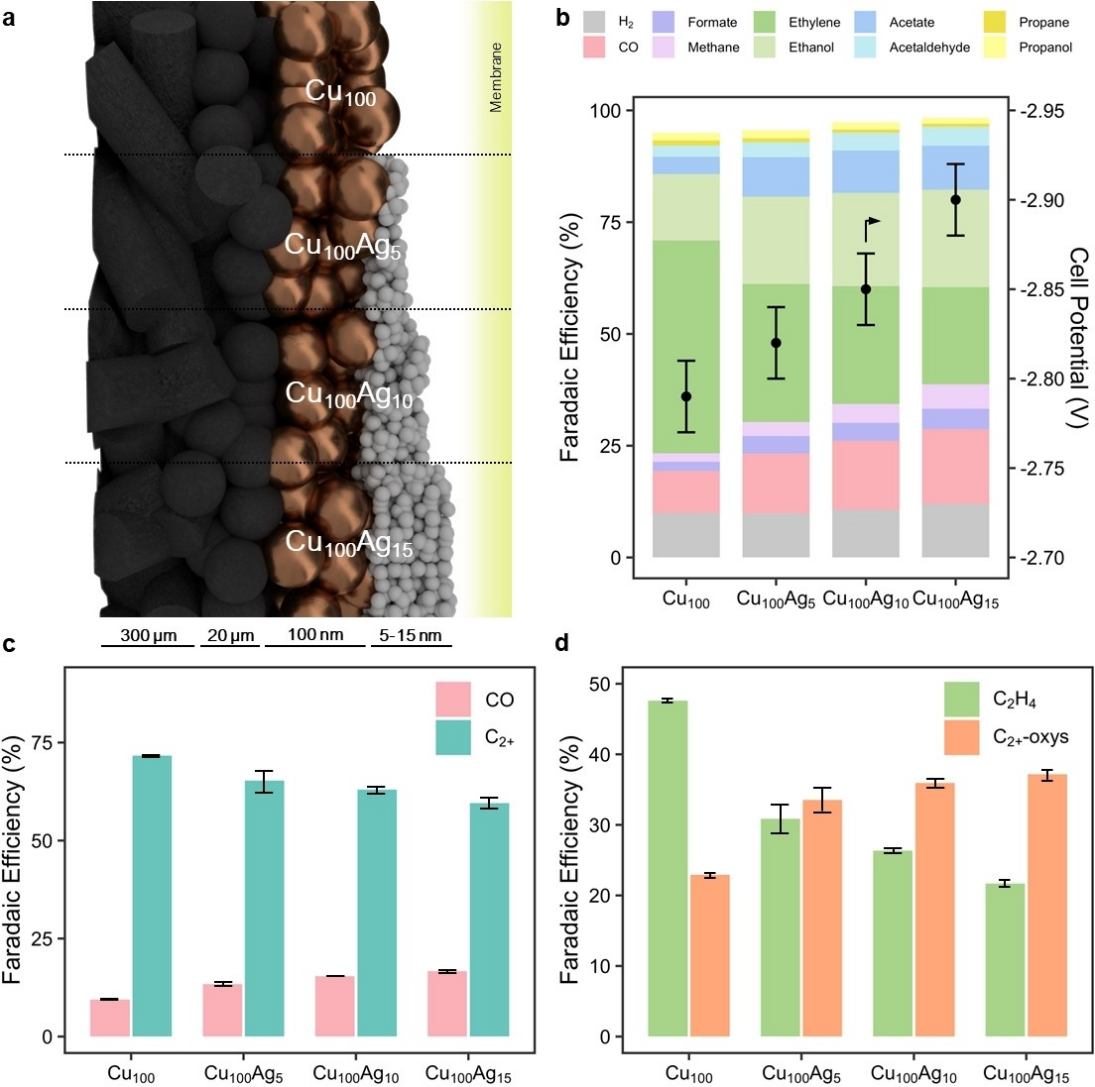
Ag overlayers do not improve C_2+_‐selectivity significantly. (**a**) Schematic representation of the four Cu_100_Ag_x_ catalysts with increasing Ag content. (**b**) Product distributions at 200 mA cm^−2^ show a decrease of ethylene with increasing Ag thickness. Simultaneously CO, ethanol, and acetate increase. (**c**) A comparison of partial current densities for CO and C_2+_ products show a gradual decline of total C_2+_ selectivity. (**d**) The ratio between multi‐carbon oxygenates (C_2+_‐oxy) and ethylene (C_2_H_4_) indicates a shift in the overall function of Cu. Displayed values are taken from triplicate experiments averaged between minute 5 and 60 at a gas product sample rate of 5 min^−1^. Error bars represent a standard deviation.

The question that arises from these observations is then: does Cu benefit at all from an ad‐layer of Ag, or is the production rate of CO on Cu sufficient on its own. To test this further and examine whether Ag can have a beneficial effect in such a configuration, we performed a new series of experiments with reduced Ag layers. Specifically, we produced and tested a 100 nm Cu layer with an added top layer of 5, 10 and 15 nm of Ag (Figure 3a in Cu_100_, Cu_100_ Ag_5_, Cu_100_ Ag_10_ and Cu_100_ Ag_15_). The layer of Ag deposited on the membrane side of the thicker Cu layer can be seen in both SEM and macroscopic images as shown in Figure S1 and S5.

Similar to the previous experiments we examined the selectivity and cell potential for the reduced Ag layers. As shown in Figure 3b, even a 5 nm thin Ag layer results in a net increase of CO, a trend that increases with greater amounts of Ag although the Faradaic efficiency is kept comparatively low versus the Cu_50_Ag_50_ case. The cell potential at constant current density also showed similar trends as those in Figure 2b, where pure copper displays a lower cell potential than tandem systems. Most interesting, though, was the fact that tandem systems did not result in increasing C_2+_‐selectivities (see Figure 3c). The increased availability of CO intermediates in the environment of Cu then did not appear to influence the relative rate of multicarbon compounds at a fixed current density.

The composition of the C_2+_ fraction, which went down from ~70 % to ~60 % upon increasing Ag content, also changed noticeably (see Figure 3d and Table S2). Where ethylene is the main product for pure copper (with a ratio of 45 %:25 % compared to the oxygenated species ethanol, acetaldehyde, acetate and propanol), the balance changes upon adding a very thin overlayer of silver. With the thickest silver overlayer, an approximate ratio of 20 %:35 % ethylene to oxygenates is reached. To explain the observed increasing oxygenate trends, we must turn our attention to the differences in micro‐environment composition for Cu_100_ vs Cu_100_ Ag_15_.

Given the orders of magnitude difference between penetration depth of CO_2(aq)_ (~5 μm)[^42^, ^43^] and the thickness of the catalyst layer (100–115 nm), we may assume a near‐to‐constant concentration of reactant CO_2(aq)_ over the depth of the catalyst. The introduction of a silver overlayer induces the excessive aqueous formation of CO, creating a steep concentration gradient towards the gas phase. We then expect the higher concentration of CO within the copper section of the tandem system results in increased oxygenate production resembling pure CO environments.[^13^, ^44^, ^45^] Further, an increased residence time of the intermediate CO can also induce a higher methane production rate, which can indeed be seen in Figure 3b.^[49]^

To further contextualize the results and assess any bulk versus atomistic effects occurring, we also performed a triplicate of experiments using gold (Au) instead of silver. These experiments again used a 100 nm Cu layer, with a 15 nm Au layer on top (Cu_100_Au_15_), and are shown in Figure S6. Here similar to the Cu_100_ Ag_15_ case we see a proportionally larger CO signal, and a higher oxygenate to ethylene ratio of ~0.5. These results would indicate that a tandem effect is occurring as hypothesized, as compared to an atomistic effect as Au is much less oxophillic than Ag. Further, the much higher CO and H_2_ signal overall as compared to the Ag case shows the greater activity of the thin Au layer, with CO FE′s increasing up to >30 %. This indicates that an even thinner layer is needed to bring CO to lower amounts.

**Figure 4:**
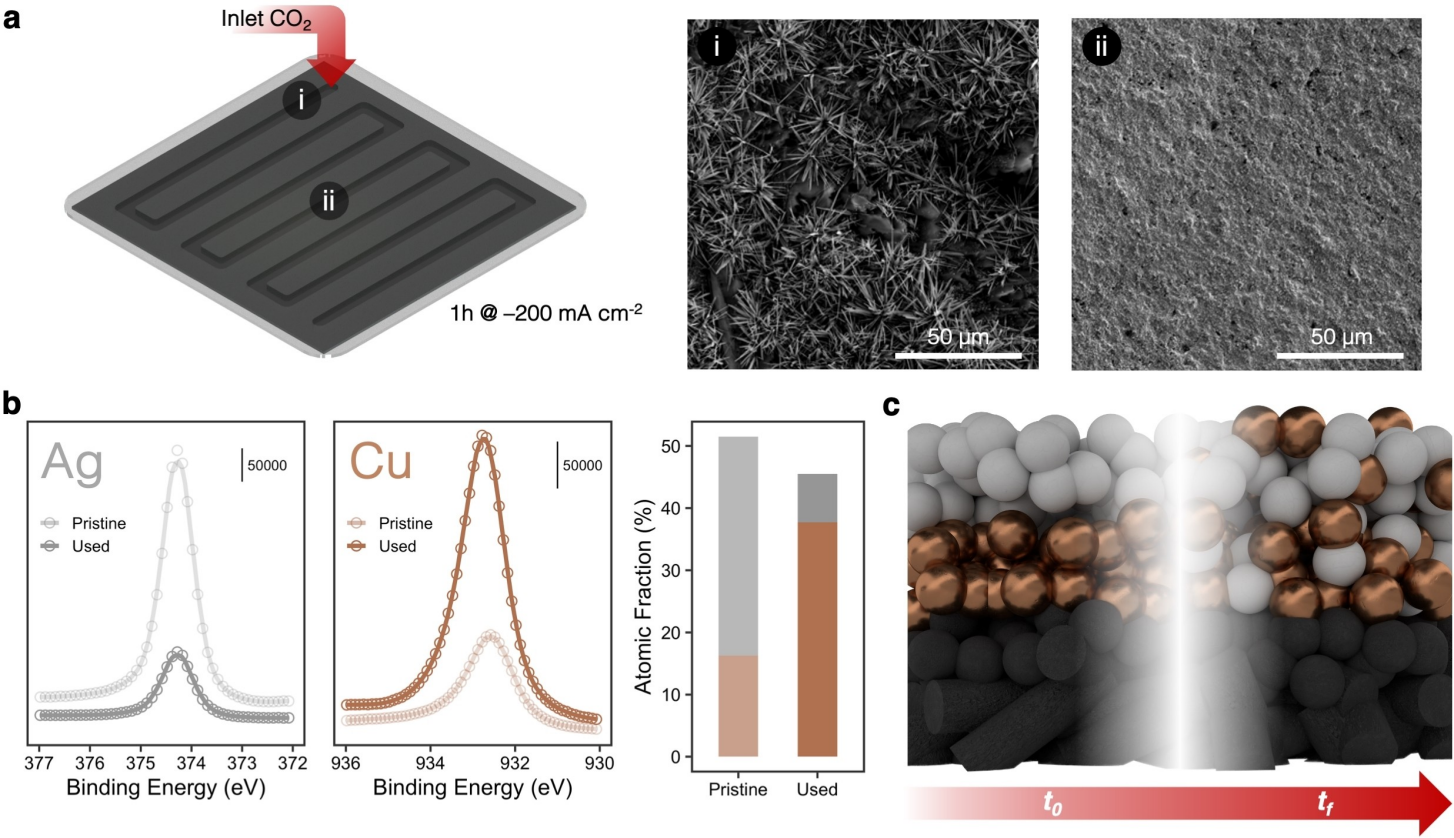
Degradation and reordering phenomena are spatially distributed in tandem catalysts. (**a**) Schematic representation of a GDE. Spots **i** and **ii** indicate analysis points at the entry and center of the GDE.s SEM‐images of spots **i** and **ii** after 1 h operation at −200 mA cm^−2^. (**b**) XPS results of a pristine and a used GDE show notable differences in surface species. The atomic fraction infers a migration of copper to the surface. (**c**) At *t_0_*, the structured layer has a defined Cu and Ag regime, whether at *t_f_* Cu has percolated through the Ag layer.

Another important consideration in tandem systems is that the stability of Cu might play a crucial role due to its tendency for surface reconstruction.33,[^47^, ^48^] Upon visual examination of the GDE surface, we noticed substantial inconsistencies in the morphology of the catalyst layer for all samples containing Cu (see Figure S7 of samples after reaction). Detailed examination using scanning electron microscopy revealed that significant restructuring of the catalyst surface mostly occurred in the upper half of the electrode closer to the CO_2_ inlet, while we observed minimal to no restructuring in the center and at the end of the flow field path (Figure 4a). Cu at the entrance half of the electrode reorganized into needle‐like clusters and, whereas the surface in the center seemed unaltered.

To assess whether the reaction affected the composition of the samples and the integrity of the layers, we conducted X‐ray photoelectron spectroscopy (XPS) analysis on both fresh and spent samples. We observed significant differences in the intensity of peaks corresponding to Ag and Cu, respectively (Figure 4b and Figure S8, Table S3). Consequently, the reaction process caused a considerable shift in the original 1 : 2 ratio of Cu to Ag to a ratio of 4 : 1 after the experiment (Figure 4b, right panel). These results indicate that the restructuring of Cu causes the two metals to become interspersed. Any layering of copper materials is then likely to be short lived unless a means for stabilizing copper is uncovered.

## Conclusions

In this study, we explored whether employing a CO‐selective catalyst (Ag) in tandem CO_2_ electrolysis could influence the composition and overall yield of C_2+_ products by conducting experiments with a series of layered Cu−Ag bulk catalysts in a zero‐gap MEA configuration. Through initial screening of bulk catalysts, we discovered that the order of deposition significantly influences the operating potential and product selectivity of the catalytic system as a whole. Specifically, we observed that the catalyst deposited closest to the gas phase exerted the most pronounced effect. However, the excessive amount of Ag initially utilized resulted in an overproduction of CO for the overall catalytic system. Upon substantial reduction of the thickness in the Ag layer, we observed a shift towards oxygenate‐type products, accompanied by a simultaneous decrease in the total CO_2_−to−C_2+_ Faradaic efficiency (FE). We attributed this behavior to the increased local concentrations of CO, which results in a product spectrum closer to that of CO reduction instead of CO_2_RR. Interestingly, despite the increased availability of CO intermediates in the environment of Cu, we still did not observe a positive influence on the total rate of formation of higher hydrocarbons, unlike recent reports that rely on much thicker catalyst layers (~4 μm).38,^[49]^ Thus, we conclude that under most scenarios a Cu catalyst is capable of generating sufficient CO to facilitate C−C coupling and does not significantly rely on the extra supplied CO under industry‐relevant high‐current density conditions.

## Author Contributions

Conceptualization: MS, Methodology: MS, Investigation: MS, Characterization: MS, HPIvM, Visualization: MS, HPIvM, Funding acquisition: TB, WS, Project administration: MS, Supervision: TB, WS, Writing – original draft: MS, HPIvM, Writing – review & editing: MS, HPIvM, NK, TB

## Conflict of Interests

The authors declare no competing interests

1

## Supporting information

As a service to our authors and readers, this journal provides supporting information supplied by the authors. Such materials are peer reviewed and may be re‐organized for online delivery, but are not copy‐edited or typeset. Technical support issues arising from supporting information (other than missing files) should be addressed to the authors.

Supporting Information

## Data Availability

The data that support the findings of this study are available from the corresponding author upon reasonable request.
