# Supplementary material for: Bulk Layering Effects of Ag and Cu for Tandem CO2 Electrolysis
Source: ChemSusChem. 2024 Dec 3;18(8):e202401769. doi: 10.1002/cssc.202401769 (PMC11997910; doi:10.1002/cssc.202401769)
Supplement: Supplementary file 1 — Supporting Information [file CSSC-18-e202401769-s001.pdf]

ChemSusChem

Supporting Information

Bulk Layering Effects of Ag and Cu for Tandem CO_2 Electrolysis

Mark Sassenburg, H. P. Iglesias van Montfort, Nikita Kolobov, Wilson A. Smith, and Thomas Burdyny*

ChemSusChem
Supplementary Information

Bulk Layering Effects of Ag and Cu for Tandem CO_2 Electrolysis

Authors: Mark Sassenburg[1], H.P. Iglesias van Montfort[1], Nikita Kolobov[1], Wilson A. Smith[1,2,3], Thomas Burdyny[1]*

Affiliations:

[1] Department of Chemical Engineering, Delft University of Technology, Delft 2629HZ, the Netherlands

[2] Department of Chemical and Biological Engineering and Renewable and Sustainable Energy Institute (RASEI), University of Colorado Boulder, Boulder, Colorado 80303, United States

[3] National Renewable Energy Laboratory, Golden, Colorado 80401, United States

*Corresponding author. Email: t.e.burdyny@tudelft.nl

This PDF file includes:

Figs. S1 to S8
Tables S1 to S3

Supplementary Figures

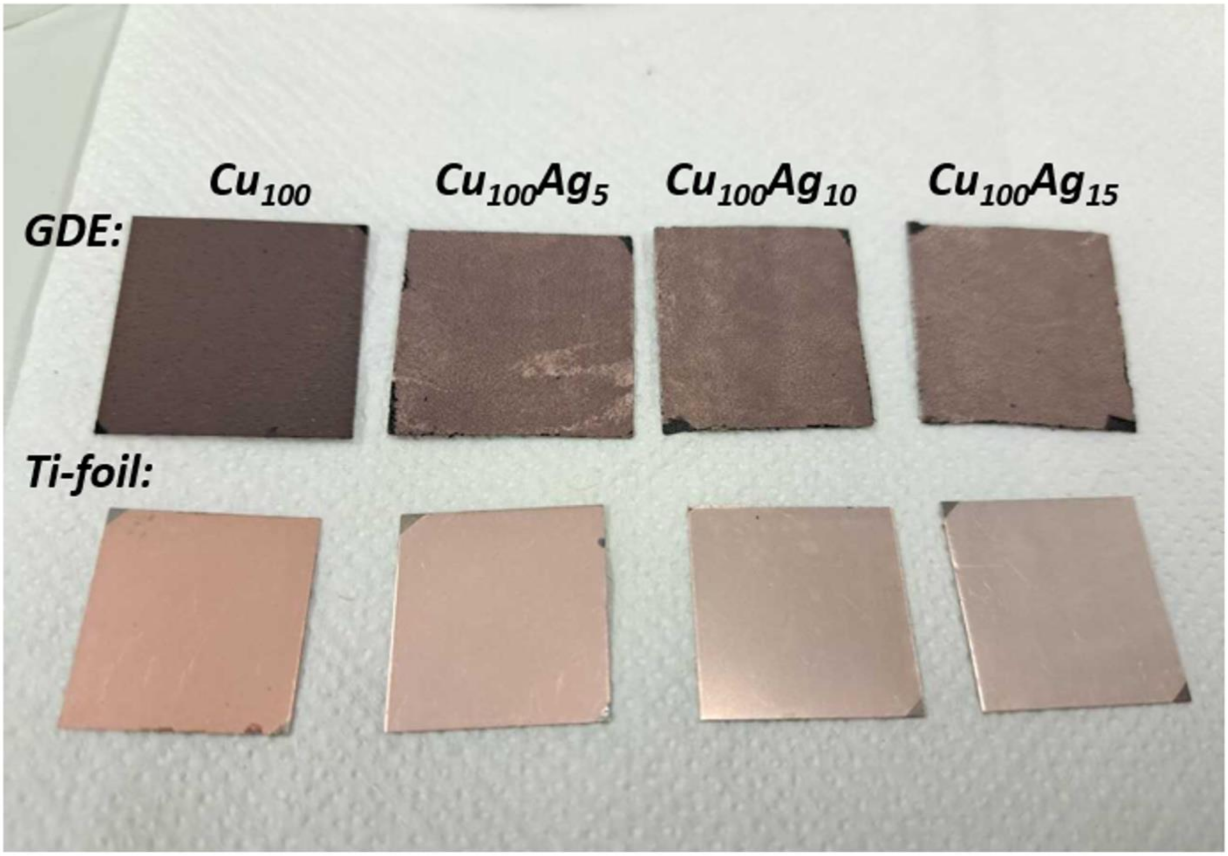

Figure S1: Sputtered copper electrodes with increasing Ag-overlayer thicknesses. A fading of the typical salmon color of Cu is visible for increasing Ag-thickness.

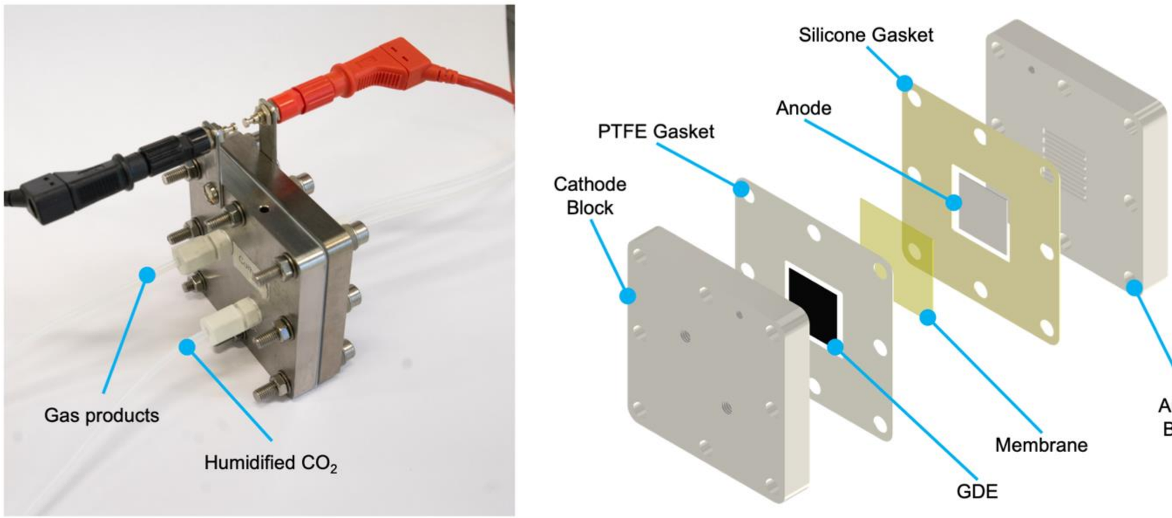

Figure S2: Zero-gap MEA cell architecture used, as described in the experimental section. Adapted from [41].

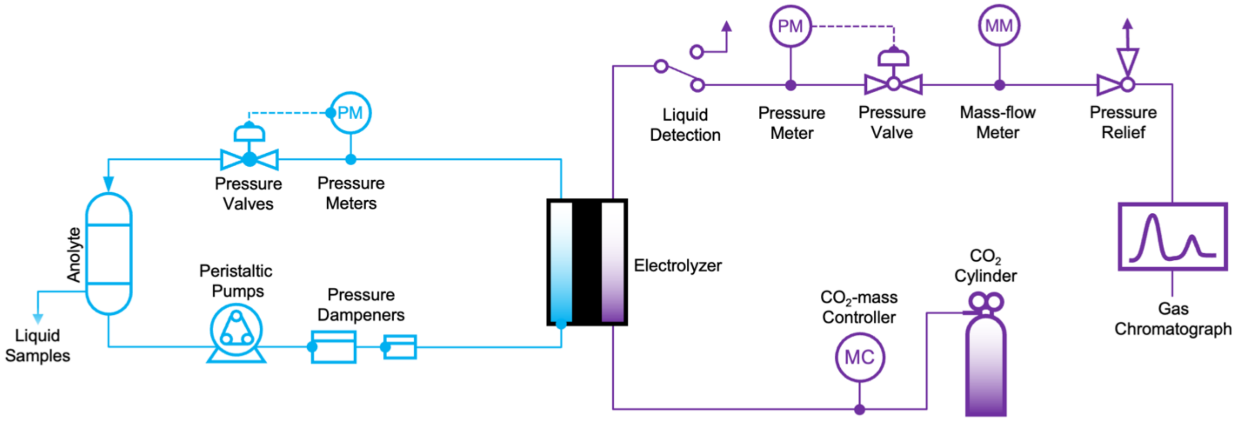

Figure S3: Bench testing setup as described in the experimental section. The liquid loop is regulated by a back-pressure regulator and pressure dampeners that sooth the periodic pressure spikes of the peristaltic pump. The gas feed is measured both at inlet and outlet, and its pressure development monitored towards analysis at a gas chromatograph.

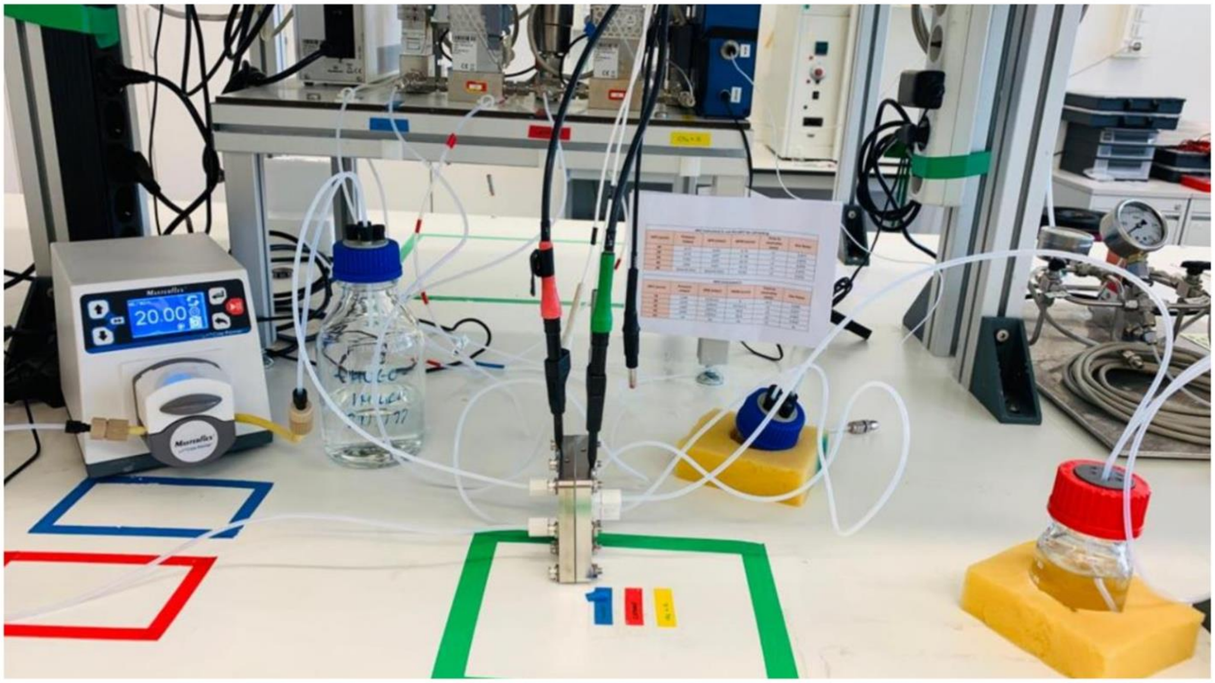

Figure S4: Picture of the lab setup as detailed in Fig. S3.

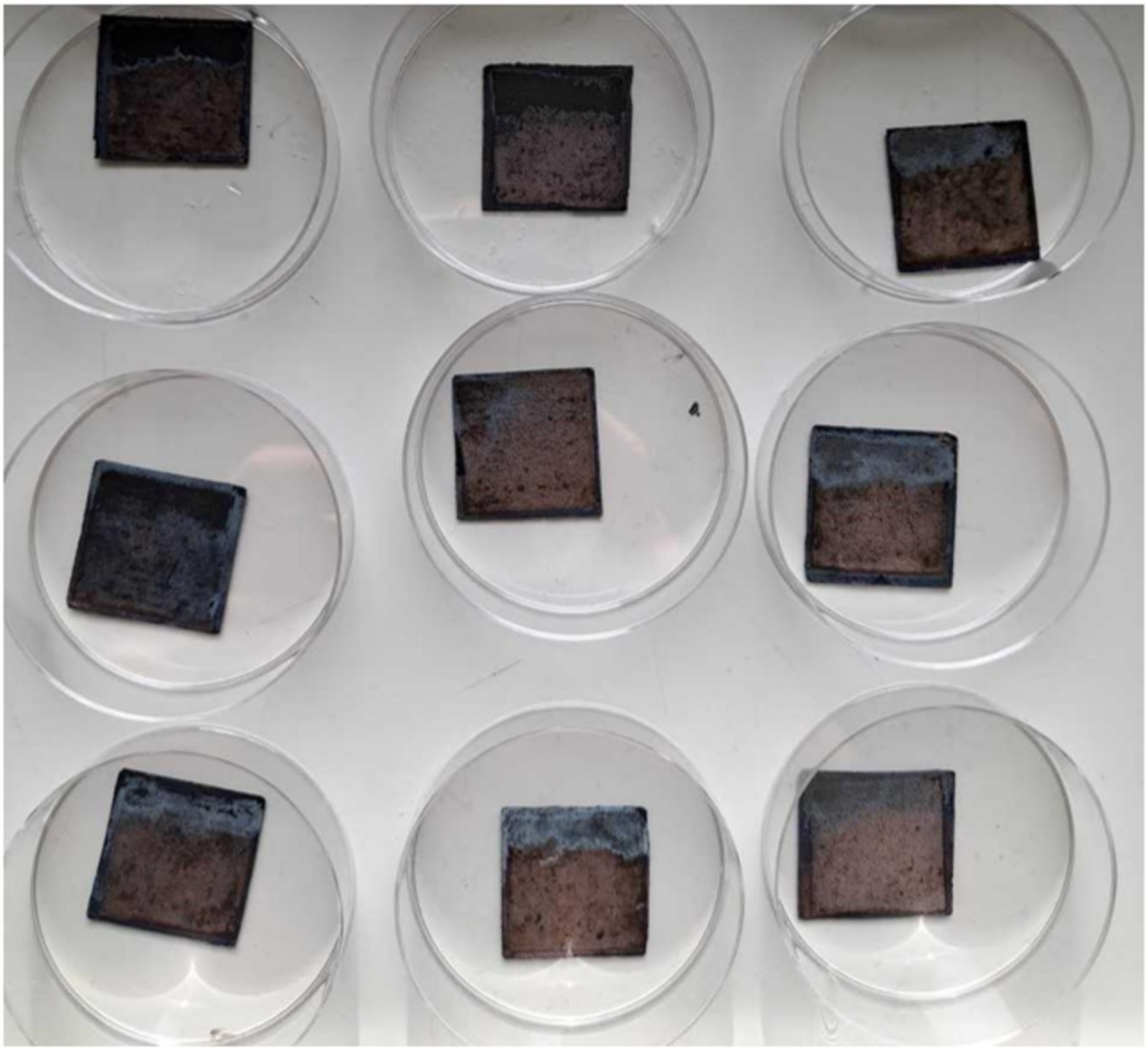

$Cu_{100}Ag_5$ $Cu_{100}Ag_{10}$ $Cu_{100}Ag_{15}$

Figure S5: Optical appearance of GDE's after chronopotentiometries at -200 mA cm^{-2} for 1 hour and increasing Ag overlayer thickness. A clear uneven salt deposition at the cathode-membrane is visible between the inlet section and the rest of the electrode.

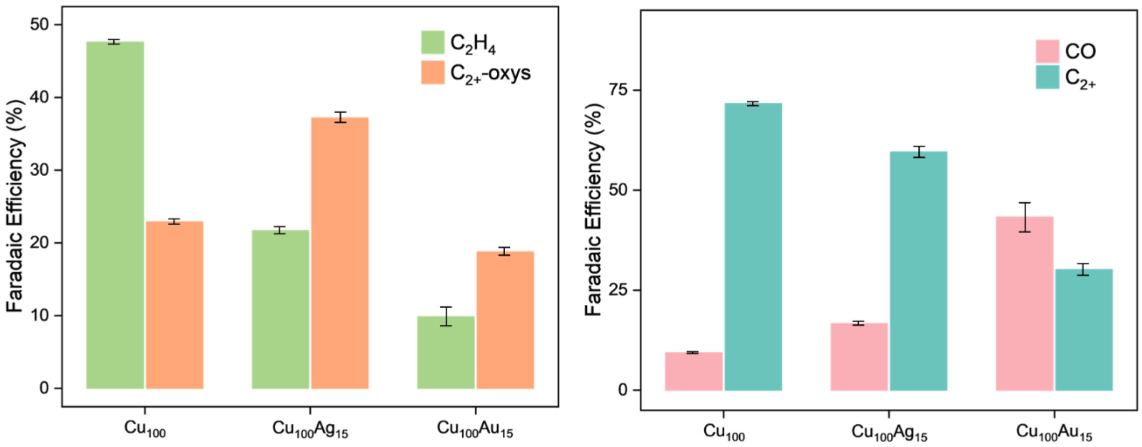

Figure S6: Comparison of Faradaic efficiencies for three different cases (Cu_{100}, $Cu_{100}Ag_{15}$ and $Cu_{100}Au_{15}$) at 200 mA cm^{-2} in a membrane electrode assembly. The subscript denotations of 100 and 15 indicate deposition thicknesses of 100 nm and 15 nm, respectively. Displayed values are taken from triplicate experiments averaged between minute 5 and 60 at a gas product sample rate of 5 min^{-1}. Error bars represent a standard deviation.

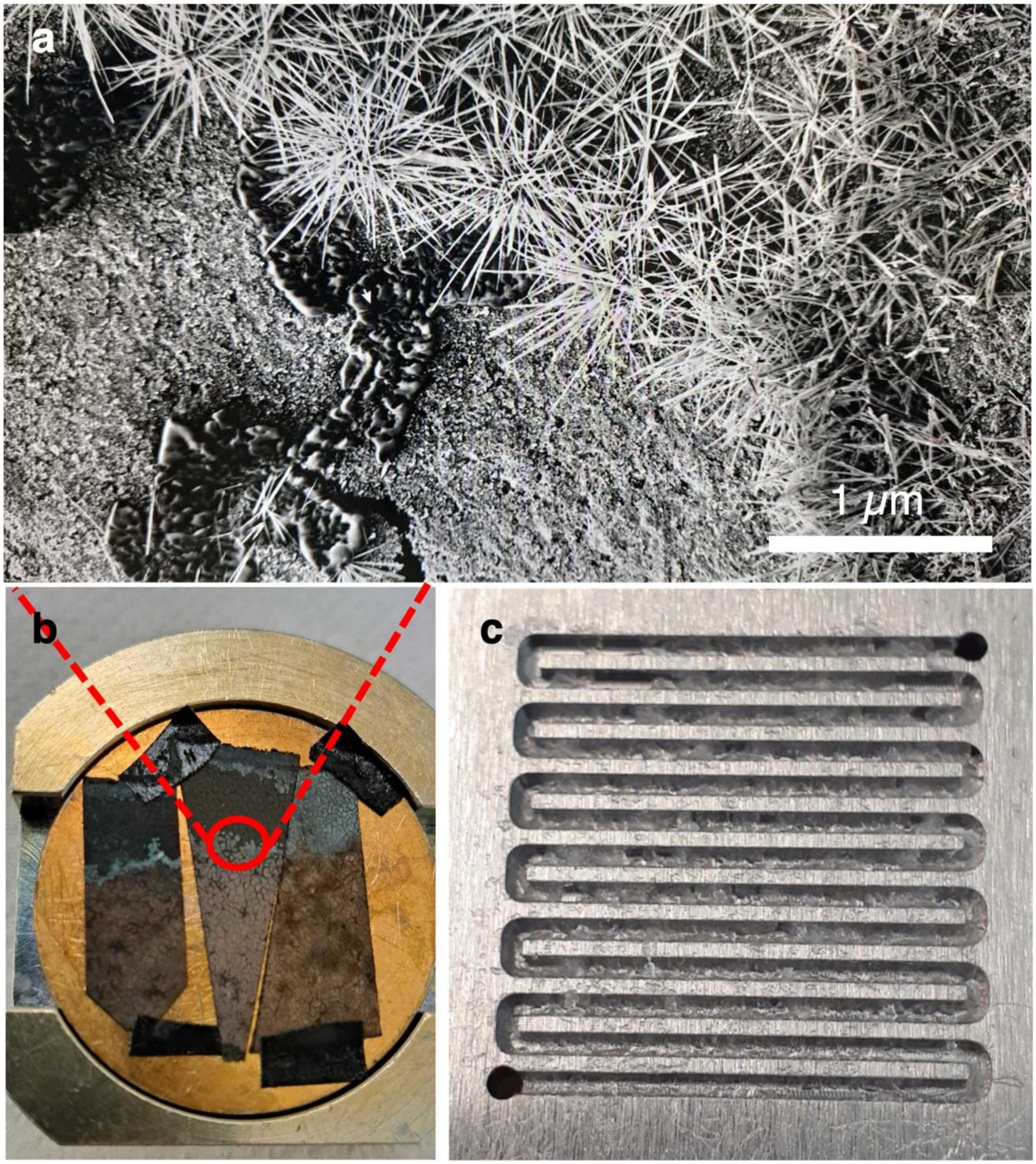

Figure S7: (a) SEM image of the interface section depicted in (b). (c) Salt formation at the gas-chamber is equal throughout the path of the gas stream (top to bottom).

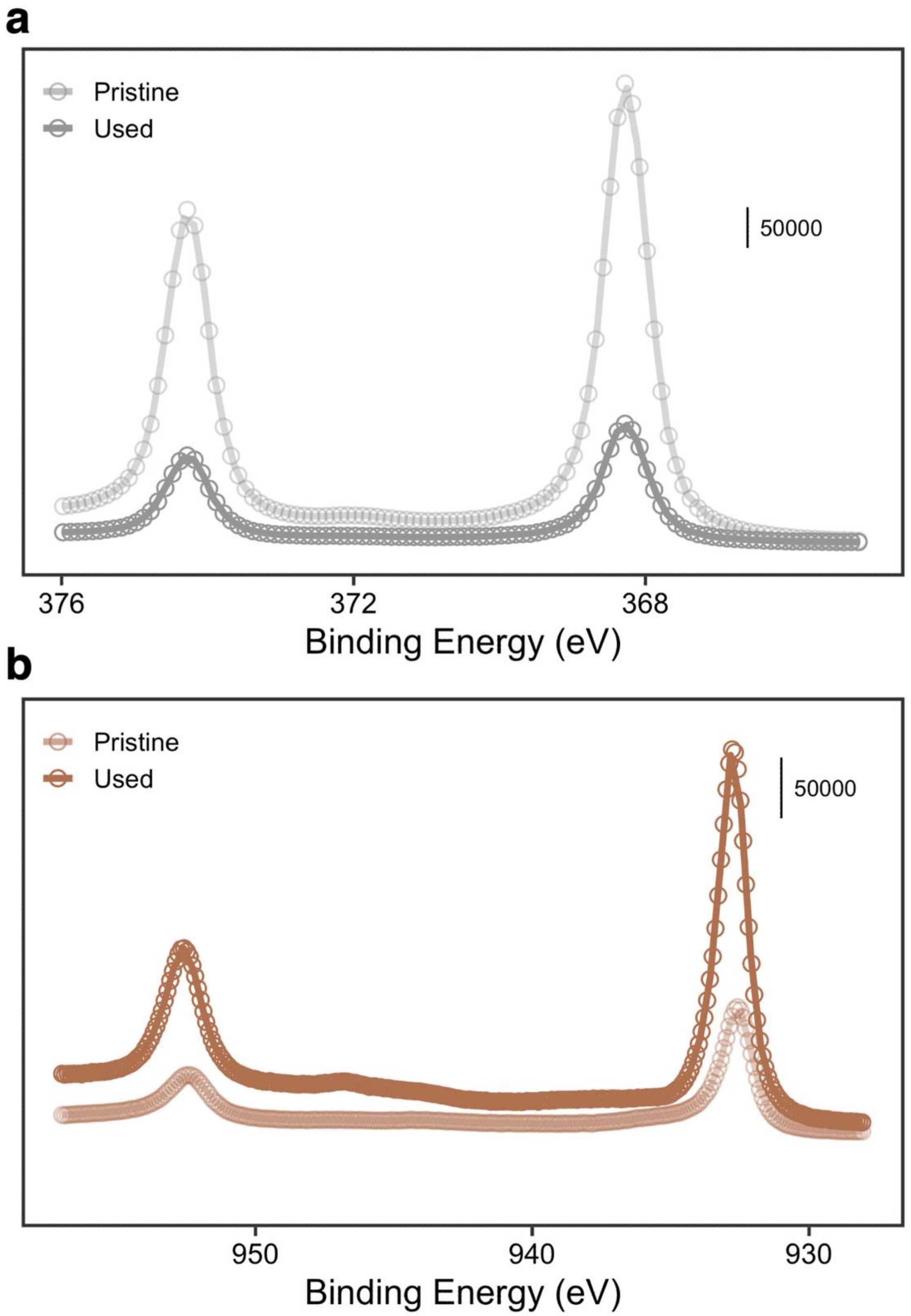

Figure S8: Fully plotted region of interests for used and pristine electrode's (a) silver and (b) copper XPS spectrograms.

Table S1

Product selectivities for different CuAg sputtered compositions, as depicted in Figure 2b. Data taken at a fixed current density of 200 mA cm^{-2}.

Faradaic Efficiency [%]

**Ag:Cu**	Hydrogen [H_2]	CO [CO]	Formate [$HCOO^-$]	Methane [CH_4]	Ethylene [C_2H_4]	Ethanol [C_2H_5OH]	Acetate [CH_3COO^-]	Acetaldehyde [C_2H_4O]	Propane [C3H8]	**E_{cell} [V]**
**Ag_{100}**	4.32	81.71	10.37	0.00	0.00	0.00	0.56	0.00	0.00	**-2.88**
**$Ag_{50}Cu_{50}$**	4.42	50.71	11.45	2.24	14.09	6.96	3.74	0.00	0.34	**-2.86**
**$Cu_{50}Ag_{50}$**	7.91	39.88	4.88	3.86	17.41	14.55	4.73	0.00	0.37	**-2.83**
**Cu_{100}**	9.95	9.41	2.01	1.92	47.61	14.87	3.85	2.52	1.16	**-2.79**

Table S2

Product selectivities for different increasing silver overlayers, as depicted in Figure 3b. Data taken at a fixed current density of 200 mA cm^{-2}.

**$CuAg_x$**	Hydrogen [H_2]	CO [CO]	Formate [$HCOO^-$]	Methane [CH_4]	Ethylene [C_2H_4]	Ethanol [C_2H_5OH]	Acetate [CH_3COO^-]	Acetaldehyde [C_2H_4O]	Propane [C3H8]	**E_{cell} [V]**
**Cu_{100}**	9.95	9.41	2.01	1.92	47.61	14.87	3.85	2.52	1.16	**-2.79**
**$CuAg_5$**	9.89	13.35	3.91	3.12	30.87	19.54	8.86	3.34	0.88	**-2.82**
**$CuAg_{10}$**	10.70	15.37	4.05	4.20	26.35	20.86	9.51	3.96	0.71	**-2.85**
**$CuAg_{15}$**	11.96	16.72	4.60	5.48	21.70	21.82	9.80	4.25	0.68	**-2.90**

Supplementary Table 3

Atomic fractions of plotted spots in Figure 4. Spot i and ii as detailed in Fig. 4.

Element	Atomic Fraction [%]	Spot	Sample
Cu	16.3	ii	Pristine
	16.3	i	Pristine
	37.7	ii	Used
	7.5	i	Used
Ag	35.2	ii	Pristine
	35.2	i	Pristine
	7.8	ii	Used
	1.91	i	Used
